# Quality of Life and Experience of Patients with Heart Failure with Preserved Ejection Fraction and Their Caregivers

**DOI:** 10.3390/jcm14134715

**Published:** 2025-07-03

**Authors:** Raül Rubio, Beatriz Palacios, Luis Varela, Martín Gutiérrez Ibañez, Selene Camargo Correa, Elena Calvo Barriuso, Nuria José, Sergi Yun Viladomat, María Teresa Soria Gómez, Esther Montero Hernández, Encarna Hidalgo, Cristina Enjuanes, Yolanda Rueda, Maite San Saturnino, Paloma Garcimartín, Jorge V. López-Ibor, Javier Segovia-Cubero, Josep ComínColet

**Affiliations:** 1 A Piece of Pie SL, 08007 Barcelona, Spain; raul@piecepie.com (R.R.); selene@piecepie.com (S.C.C.); 2 Medical Department, AstraZeneca Farmacéutica Spain SA, 28050 Madrid, Spain; beatriz.palacios@astrazeneca.com (B.P.); luis.varela1@astrazeneca.com (L.V.); martin.gutierrez@astrazeneca.com (M.G.I.); 3 Department of Cardiology and Heart Failure Program, Bellvitge University Hospital, 08907 Barcelona, Spain; ebarriuso@bellvitgehospital.cat (E.C.B.); njose@bellvitgehospital.cat (N.J.); syunvi@gmail.com (S.Y.V.); encarna.hidalgo@gmail.com (E.H.); cristinaenjuanes@gmail.com (C.E.); 4 Bio-Heart Cardiovascular Diseases Research Group, Bellvitge Institute for Biomedical Research (IDIBELL), 08907 Barcelona, Spain; 5 Heart Failure Unit, Puerta de Hierro University Hospital, 28222 Madrid, Spain; t.soriagomez@gmail.com (M.T.S.G.); esthermhdez@hotmail.com (E.M.H.); jvazlopez@gmail.com (J.V.L.-I.); jsecu@telefonica.net (J.S.-C.); 6 CIBER of Cardiovascular Diseases, Carlos III Health Institute, 28029 Madrid, Spain; 7 CardioAlianza, 08021 Barcelona, Spain; hola@cardioalianza.org (Y.R.);; 8 Outpatients Clinics, Hospital del Mar, 08003 Barcelona, Spain; pgarcimartin@hmar.cat; 9 Department of Biomedical Research in Heart Diseases, Hospital del Mar Institute for Medical Research, 08003 Barcelona, Spain; 10 Department of Clinical Sciences, School of Medicine, University of Barcelona, 08036 Barcelona, Spain

**Keywords:** health lived experiences, health outcomes, heart health, power, empowerment, practice guidelines, medical anthropology

## Abstract

**Background/Objectives**: Evidence of patient experiences with heart failure with preserved ejection fraction (HFpEF) and disease impact on quality of life (QoL) is scarce. This study explored perceived impacts on QoL and healthcare experiences of HFpEF patients and their caregivers. **Methods**: This was a mixed-methods study with HFpEF patients, ≥40 years, New York Heart Association functional classes I-IV in Spain. Qualitative data were collected through semi-structured interviews with patients (*n* = 19) and caregivers (*n* = 17). The EuroQoL 5D-5L, Patient Global Impression of Severity, and Kansas City Cardiomyopathy Questionnaire were used to collect QoL measures. **Results**: The themes were as follows. (1) Impact of HFpEF on QoL; (2) new roles of informal caregiving; and (3) the increasing value of multidisciplinary care. Qualitative data were supported by a trend of worsening QoL on quantitative measures as HF progressed, despite quantitative measures not fully capturing the burden. Qualitative data further captured discrepancies of QoL perceptions. **Conclusions**: The impact of HFpEF on patients and their caregivers was similar to the HFrEF population’s. Insights from discrepancies between PROMs data and interviews could help with tailoring QoL questionnaires to capture the broader impact of HFpEF, identify unmet needs, and customize care.

## 1. Introduction

Even with advances in the identification of categories and management approaches, heart failure (HF) continues to stand as a leading cause of death, disabilities, hospitalizations, and readmissions [1], accounting for a large proportion of developed countries’ national health expenditure [2,3,4]. HF can severely impact patients’ quality of life (QoL) by reducing their autonomous ability to undertake certain daily activities and impacting psychosocial and economical capacities. For instance, HF was reported to create a sense of isolation, precluding active lifestyles and creating a loss of agency in patients with HF with reduced ejection fraction (HFrEF). Other studies have shown that HF patients experience depression, anxiety, and an overall decreased QoL [5].

However, qualitative studies of the experiences of patients with preserved HF (HFpEF) and the impact of the disease on their QoL are much more scarce [6]. Different patient populations have been described for HFrEF—characterized by left ventricular ejection fraction (LVEF) of ≤40%—and HFpEF—characterized by LVEF of ≥50%—at an epidemiological level [7]. For instance, HFpEF patients are usually older, most commonly women, and with a higher prevalence of obesity, anemia, atrial fibrillation, and hypertension [7]. They have also been found to have lower levels of serum natriuretic peptides [7]. In comparison with HFrEF, patients with HFpEF drink more alcohol and smoke less, and they tend to have chronic pulmonary disease, chronic kidney disease, and valvular heart disease more often [8]. These distinctions may differently impact disease experience and QoL in patients with HFrEF and HFpEF. Consequently, it is important to investigate not only the experiences of patients with HFrEF but also those of patients with HFpEF, as they account for approximately 50% of the HF population [8].

Despite many studies reporting biomedical perspectives, little is known about patient and caregiver perspectives [9,10,11]. However, patient experiences are essential in order to develop a comprehensive appreciation of the impact of HF on patients’ lives and enhance outcomes [10]. Furthermore, it has been suggested that greater caregiver involvement may improve patients’ health outcomes [12]. Consequently, the inclusion of caregivers’ perspectives is crucial.

In research settings and clinical practice, patient-reported outcome measures (PROMs) contribute to patient management of heart conditions by providing complementary measures to traditional standard biomedical outcomes [13]. PROMs involve questionnaires assessing QoL, including both generic instruments (e.g., EuroQoL 5D-5L (EQ-5D-5L) and the disease-specific Kansas City Cardiomyopathy Questionnaire (KCCQ) [14]. Also, patients’ experiences with healthcare and sociosanitary systems are important to improve the provision of chronic healthcare. In this sense, the Instrument for Evaluation of the Experience of Chronic Patients (IEXPAC) focuses on interactions between patients and social/healthcare providers [15].

However, in the field of HF research, there are limited studies that explore how PROMs differ from real-world disease experiences. Moreover, most research on HF and PROMs is focused on patients with HFrEF [5,16,17]. A recent observational study that aimed to understand HFrEF patients’ experiences revealed an existing gap between PROMs and patients’ narratives; patients did not always articulate health-related status and QoL in completing the questionnaires, but they did so during the interviews, indicating that QoL is subjective.

Our study aimed to bridge this gap for patients with HFpEF by enriching our knowledge of this population’s QoL from the perspective of patients and their caregivers. More specifically, the study had two aims. First, this study aimed to address the perceived impact of HFpEF on QoL and patients’ healthcare experiences and to gather information on caregiver perspectives. Second, the study aimed to examine agreements and discrepancies between the descriptive quantitative data gathered through PROMs and the qualitative data gathered through semi-structured interviews.

## 2. Materials and Methods

### 2.1. Study Design

This was a cross-sectional, observational, multi-center, and mixed-methods study with 19 HFpEF patients and 17 caregivers. We used an embedded mixed-methods approach involving the parallel use of in-depth interviews with PROMs for the assessment of health status (EQ-5D-5L, PGIS, KCCQ, and IEXPAC). In particular, quantitative and qualitative data were collected during the same interview with each participant. The main purpose of this method was to support the qualitative data to observe if quantitative data contradicted or supported the primary data [18]. Rather than seeking corroboration of results from different data sources, this approach intended to highlight the complementarity of qualitative data and PROMs [18,19].

Patients were recruited from two university hospitals in Spain between July and December 2021. These medical centers relied on integrated units of HF composed of multidisciplinary teams providing clinical care for the prevention, diagnosis, and treatment of HF, similar to international programs [20,21,22]. The two participating hospitals were located in areas with different socioeconomic contexts in two different cities in order to achieve sample variation among participant patients and caregivers. Participants were treated and followed up by specialized, multidisciplinary units (cardiology, internal medicine, advanced practice nursing). Any decision associated with the management of HF was left to the judgement of the patient’s physician.

### 2.2. Sample and Recruitment

The sample size for this study was 19 patients and 17 caregivers. According to the existing literature, a sample of this size is sufficient for empirical saturation, as statistical analyses of diminishing returns indicate that most new information is typically captured in the first five to twelve interviews [23,24,25,26,27]. Caregivers were included as participants to paint a broader, richer picture of HFpEF’s social impact. Caregivers’ perspectives also provided more in-depth information on how patients make sense of the disease during diagnosis, treatment, and follow-up.

Patients were recruited consecutively by HCPs during follow-up visits at the two participating clinical sites after they had been informed of the study’s purposes and participation terms and conditions. The diagnosis of patients who participated was validated using clinician-provided COD (Confirmation of Diagnosis), typically regarded as the gold standard for providing complete certainty that patients have the disease in question. The candidates were assessed for eligibility according to the following inclusion criteria. (a) A prior diagnosis of HF (documented signs/symptoms in the previous 12 months or on diuretics) with pEF/LVEF of 50% or greater [28,29] and at least two of the following: (i) hospitalization with a primary diagnosis of HF at discharge, (ii) evidence of structural heart disease (e.g., left ventricular hypertrophy, left atrial enlargement) on echocardiography and/or cardiac magnetic resonance, (iii) NT-pro BNP ≥ 300 pg/mL (≥600 pg/mL for ongoing atrial fibrillation/flutter); (b) New York Heart Association (NYHA) functional class I to IV following verification with the Goldman scale; and (c) aged ≥ 40 years at the time of the study. HF was classified according to the European Society of Cardiology (ESC) 2021 Guidelines on the basis of LVEF [30]. Exclusion criteria for patients were (a) unable to complete the PROMs and (b) hospitalization at inclusion.

Patients were given the opportunity to accept having their caregiver interviewed by opting in through the written consent form, and 17 accepted. There were no exclusion criteria for caregivers. They were consecutively recruited based on their willingness to participate, and they provided verbal informed consent prior to the interview, as approved by the ethics committee. This procedure was adopted to facilitate their participation.

### 2.3. Data Collection

Background and medical history were collected from medical records. Patients’ social characteristics, such as gender, education, lifestyle, and caregiver support, were considered in the analysis, as they can contribute to the prevalence of cardiovascular disease and variations in health status over time [17,31,32]. Patient and caregiver data were collected through semi-structured interviews and self-administration of PROMs. Patients responded to the four selected PROMs, while caregivers only responded to the IEXPAC questionnaire. Data from PROMs were collected at the same time point of the interview.

The semi-structured interview guide was developed by the study team, which comprised social scientists, for specific use in this study. Interviews were 90 min long and conducted at patients’ homes. One caregiver was present in 17 of the patients’ interviews and participated as well. All interviews were audio-recorded, transcribed verbatim, and analyzed using thematic analysis. The interviewers had no previous relationships with participants. The interview guide was designed in a way that allowed the interviewer to build rapport with the patient so that they felt at ease and shared their emotions, feelings, and experiences around HF. The main topics discussed concerned the patients’ contexts, the meanings they attached to QoL, their experiences with the disease, and their relationships with HCPs. Prior to home interviews and on the same day, the Spanish validated versions of the EQ-5D-5L [33], Patient Global Impression of Severity (PGIS) [34], KCCQ [14], and IEXPAC [15,35] were administered. Participants completed the PROMs in front of the ethnographers but without their intervention.

The EQ-5D-5L is a robust, validated, generic instrument to self-rate general health, physical, and psychosocial dimensions in numerous disease areas, inclusive of HFrEF [5,36,37,38]. The KCCQ is a validated self-administered HF-specific instrument that is useful to more precisely stratify patients and provide tailored approaches to care plans [5,39,40]. The PGIS is a global index to rate patient perceptions of the overall current severity of HF symptoms that is holistic and easy to use, correlates with clinical outcomes, and has established minimal clinically important differences [5,41,42]. The IEXPAC questionnaire is a validated, self-administered tool assessing patients’ and caregivers’ experiences with chronic illnesses, which, when complemented with other specific HF measurements, can uncover unmet needs [5,43]. Therefore, the PROMs chosen for this study have been shown to be robust measures of QoL for HFrEF and people living with chronic illnesses, and, when complemented by semi-structured interviews, they may provide a triangulated, holistic view of patients’ and caregivers’ experiences with HFrEF [5,37,43].

### 2.4. Data Analysis

All data were analyzed thematically by two different researchers, who independently reviewed data to resolve disagreements.

#### 2.4.1. Qualitative

Data were analyzed through inductive or data-driven thematic analysis to understand participants’ experiences with the disease [44,45]. Using this approach allowed themes to emerge organically. Field researchers performed thematic analysis as suggested in the literature, as follows [44]: (a) reviewing interviews’ transcripts/fieldnotes; (b) identifying main topics per interview domain; (c) codifying text to identify current/new domains; (d) reviewing interviewees’ PROM scores; (e) comparing PROM scores with fieldwork; and (f) synthesizing repetitive patterns.

#### 2.4.2. Mixed-Methods Analysis

**Figure 1:**
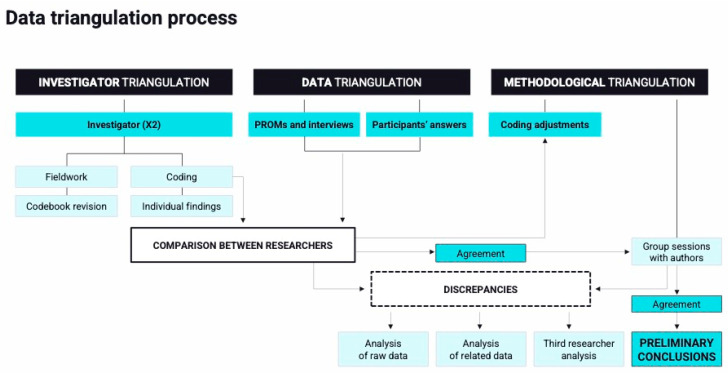
Data triangulation process.

Data triangulation was used to integrate qualitative information collected by each field researcher individually and in joint sessions. Three types of triangulation were used: (1) investigator, (2) data, and (3) methodological triangulation [46]. Findings from each field researcher were compared to develop a deeper understanding of how each researcher viewed the data. Preliminary results were discussed with the broader team of authors in joint analysis sessions to reach preliminary conclusions. Investigators compared the answers from respondents (patients and caregivers) separately to identify areas of agreement and disagreement over the main themes. During analysis sessions, discrepancies between the researchers were discussed, and raw data were examined to reach a consensus. In cases of differing opinions, additional analysis was developed considering other data that were relevant to the topic being discussed, and a third researcher joined the discussion until a common view was achieved. The comparison of PROMs and semi-structured interviews’ data served to cross-validate results and reduce interpretive bias. Additionally, the codebook used for analysis was iteratively revised and refined by both researchers throughout fieldwork, ensuring a more nuanced and rigorous interpretation of the findings. A diagram of this process can be found in Figure 1.

The described analytical process aimed at reaching theoretical saturation, where no new dimensions or patterns emerged during joint sessions.

## 3. Results

### 3.1. Participants’ Characteristics

The study included 19 patients. Their demographic and clinical characteristics are summarized in Table 1, and their main social characteristics are in Table 2. The mean (SD) age of the patient sample was 80 (7) years (range 67–92), with 58% women. The mean ejection fraction (EF) was 64% (7.3%) (range 50–78%), and the average time since diagnosis of HF was 3.3 years.

Seventeen caregivers were also included in the study, of which 77% were women. Fifty-four percent were aged ≥ 65 years, while 46% were <65 years old. Informal caregivers of HFpEF patients were often spouses (54%) or children (46%), particularly for widows or single participants. Children caregivers were predominantly female (85%) (Table 2).

### 3.2. Qualitative Findings

Three main themes were identified after thematic analysis, and they are described below. A summary of themes and illustrative quotations can be found in Table 3.

#### 3.2.1. Theme 1: Impact of HF on QoL

The first theme regards the effect of HF on physical and psychosocial outcomes for patients. Within this theme, three subthemes were identified.

(a) Fear of physical/psychological decline/fast progression

Physical decline was often described as the inability to conduct everyday activities, such as dressing, bathing, or working in the garden. The fear of falling due to equilibrium issues was also frequently mentioned by participants, who reported having difficulties walking and experiencing general mobility issues. In numerous accounts, the rapid progression of the disease was mentioned, giving participants a strong sense of losing their capabilities over time. On the psychological side, the possibility of having invasive medical interventions, health emergencies, or frequent hospitalizations was among the main concerns. Several individuals reported having a sense of impending mortality, sometimes described as “being dead while alive.”

(b) Loss of agency

The inability to travel or go on holiday was among the main limitations participants reported. The possibility of having a medical emergency away from home or the previously mentioned physical decline were the barriers they identified as also being the reason for their fear of being home alone, especially at night. These elements gave participants a strong feeling of being dependent on others, lacking autonomy, and being a burden to their loved ones. All participants had a strong desire for independence, with higher NYHA classes associated with feelings of worthlessness, loneliness, and a lack of agency in their care. Additionally, this group tended to define QoL as being independent and impairment-free.

(c) Sense of isolation

A sense of isolation was described as a result of physical weakness and limitations. Many participants explained that they were not visiting friends due to a lack of energy. The fact that they could not engage in social activities (e.g., walking) or that they had a sense of fear of experiencing health problems away from home was also a limitation for social life. The context of COVID-19 aggravated this feeling of isolation, as social interactions were less frequent in order to avoid infection. All participants stressed the importance of having their families’ support and being accompanied by them. At the time of the interviews, COVID-19 was no longer having a significant impact on medical follow-up. Nevertheless, patients reported feeling anxious and distressed during the first year of the pandemic, as they feared their condition might worsen due to the lack of regular medical visits.

Although the mentioned experiences were observed across all NYHA functional classes for newly diagnosed and long-term patients, two contrasts were found. Younger patients and patients from lower NYHA classes understood the disease as emotionally challenging and actively sought medical discharge. They expressed anxiety towards clinical encounters and showed concern regarding increasing deterioration of their QoL. They usually defined QoL as what they used to do in their immediate past.

Conversely, older patients and patients from higher NYHA classes experienced the disease as part of a suboptimal aging process and having multiple comorbidities. Older patients (>77 years old) expressed anxiety, depression, and extreme concerns regarding physical impairment and social isolation. Further details about socioeconomic factors in heart failure patients with reduced LVEF have been published in precedent work [6].

#### 3.2.2. Theme 2: The New Role of Informal Caregiving

Caregivers experienced increasing demands for care and transitions to new supporting roles and felt that patients’ aging processes accelerated with the condition. They expressed being ill-prepared to confront it alone, especially in higher NYHA classes and emergency situations. This provoked feelings of stress and anxiety in caregivers, especially when dealing with the health system or when the patient had serious health issues. Most caregivers felt a deep sense of responsibility towards monitoring the health and well-being of patients. They were usually in charge of managing medication administration, dietary needs, and patients’ mobility limitations while providing emotional support. Caregivers would appreciate receiving more guidance about how to deal with patients’ necessities at home. Support from health professionals was identified as a facilitator to enhance caregivers’ capacity and confidence to manage the health of patients.

Gender differences in caregiving by spouses arose whereby male patients were highly dependent on being taken care of by their wives, while female patients relying on their husbands were left to fend for themselves. When asked to describe what was the husband’s supporting role, many female patients and their spouses cited carrying heavy objects, such as shopping bags, and driving to and from appointments. Instead, when asked to describe what was the wife’s supporting role, many male patients and their spouses cited taking care of the medication, coordinating medical appointments, managing information about disease progression, making necessary changes to daily activities and routines, cooking, cleaning, and caring for the overall wellbeing of the husband. Thus, the burden of caregiving was generally heavier for wives than for husbands.

In several cases, especially when patients were widowed, it was their children who took on the main caregiver role. The tasks children caregivers took on were related to managing the patients’ medical condition, such as understanding and relating medical information to patients and organizing and accompanying them to medical appointments. Patients expressed feeling more of a burden when their caregivers were their children instead of when the caregivers were their spouses.

Data collected in both the interviews and IEXPAC questionnaires (Table 3 and Table 4) showed that caregivers were more critical regarding the health status and care process of the patients than patients themselves, including concerns about communication and care coordination. They were also more prone to emphasize personal and family costs of caring for a patient with HFpEF (e.g., new working arrangements, caring tasks based on geographical proximity or personal/financial means).

#### 3.2.3. Theme 3: The Increasing Value of Multidisciplinary Care

The third theme identified was the increasing value of multidisciplinary care, which highlighted that additional support from healthcare teams resulted in increased patient QoL. Overall, the relationship with HCPs was characterized by mixed feelings, highlighting a gap between primary and specialized care, as detailed below.

Among participants, there was a feeling of lack of coordination between the hospital and primary care, especially regarding communication issues between the different professionals involved in their care. Within this category, three sub-themes emerged (Table 3).

(a) New relationships with primary care (PC) physician

Patients described dissatisfaction concerning the relationship with their PC physician. They often felt that these professionals were inattentive and unresponsive to their needs. They expressed disappointment at the lack of availability and personal connection with these professionals. Communication difficulties were also common due to meeting different substitutes during medical visits. This situation entailed reduced interactions with their main PC and reinforced patients’ feelings of being abandoned.

(b) Provision of specialized support from HCPs

A new sense of engagement was observed concerning the specialized care units. Cardiology professionals were usually described as attentive, comprehensive, and highly professional. Patients felt supported by these professionals and described hospital visits as a positive experience. Participants felt that specialists took special care of them, which reinforced a feeling of agency towards the disease. Despite these positive aspects, some patients declared they wished to receive more direct answers from HCPs and more detailed explanations about the proposed treatments.

(c) Provision of nursing care by a registered nurse

The role of nurses in patient care was one of the main topics discussed across all interviews. Regular contact with these professionals provided guidance and reassurance to participants. Follow-up visits were usually made by phone every week or every two weeks and consisted of monitoring patients’ health status, giving lifestyle recommendations, and providing information to better understand the illness. Nurses were also perceived as a leading figure in coordinating patients’ medical follow-up among the different professionals and providing valuable guidance through health issues.

### 3.3. Descriptive Quantitative Data

PROM results are presented in Table 4. Regarding EQ-5D-5L, 10% of NYHA I/II patients reported severe/extreme problems with mobility, self-care, and usual activities, and 20% reported severe/extreme problems with pain/discomfort. In contrast, severe/extreme problems were common in NHYA III-IV patients, with 22% reporting moderate/extreme problems with self-care, pain/discomfort, and anxiety/depression, 33% mobility, and 44% usual activities. The mean value index (SD) and mean EQ-VAS (SD) were 0.68 (0.26) and 74 (13.56) in NYHA I/II patients and 0.34 (0.46) and 51.6 (23.4) in NHYA III/IV patients (higher scores indicate better health-related QoL).

Reporting from the KCCQ, the mean (SD) KCCQ clinical summary score was 53.49 (26.88), and the mean (SD) KCCQ overall summary score was 59.91 (28.90), with higher scores indicating fewer symptoms and better function/QoL. NHYA I/II patients reported fair to good/excellent health status across all specific sub-scales, whereas NHYA class III/IV patients reported poor to fair problems across most domains. Moreover, older patients and those with higher NYHA functional classes reported concerns related to physical impairment and social isolation. This was reflected in the descriptive quantitative data in the KCCQ physical and social limitation scores.

From the IEXPAC, mean (SD) overall IEXPAC scores with 11 items for patients and 12 items for caregivers were 6.6 (1.4) and 5.5 (2.5), respectively. Mean (SD) scores for conditional questions related to situations that are common to this population group were 6.6 (2.5) for patients and 6.5 (3) for caregivers. Higher scores indicate a better experience with health and social integrated care (10 = maximum).

**Table 3:** Summary of thematic domains and sub-themes.

Thematic Domains	Sub-Themes	Verbatim Code	Nyha Class	Examples of Verbatim Quotations
**Domain 1. The impact of HFpEF on QoL**				
Fear of decline and progression		101	I	If I had to live my whole life with this, I’d feel really bad. I hope it gets better.
		101	I	Quality of life for me means being strong and taking good care of myself to help my family in whatever way I can, and my children and grandchildren.
		201	II	Since I was admitted I feel that I have not gotten back on my feet.
		203	II	He has to begin to accept that s/he is getting older, that he has hit a slump with the heart thing. That part is hard for him to accept. [Caregiver]
		303	III	Today for example I feel fine, tomorrow I don’t know. There are days that I feel very low energy, without motivation. It’s not that I’m depressed, but just mad at myself because I’m not feeling well. There are days when I don’t do anything, I can’t go anywhere very far. I don’t even feel like reading because I get tired.
		304	III	The night before an appointment with the doctor, I don’t sleep. I have been hospitalized so many times unexpectedly that I am always afraid to go, in case they see that I am not well and I have to be hospitalized.
		401	IV	It has gotten worse and worse, now my daughters have sent me a lady to come and help me at home. For the past year I can no longer do things alone, neither get dressed nor clean myself.
Loss of agency		103	I	We used to travel a lot around the country and abroad, but now this limits us. If we go on vacation or on a daytrip, we do it around here. We get scared. We no longer want to be too far from the hospital. [Caregiver]
		205	II	Quality of life is being independent. Walking, being able to drink beer with my friends and do everyday things without getting tired.
		204	II	If I’m walking down the street and I feel a little sick or short of breath, I put the wheelchair on the turbo setting and come home.
		305	III	For me, quality of life it is being independent. I suffer because now I am not able to go to the supermarket, make my bed, prepare meals or clean the house.
		301	III	How can I have any quality of life? My legs have been giving me a lot of trouble for several years now. I have rheumatism and osteoarthritis in my knees and it’s never going to get cured. No doctor has cured my osteoarthritis, and with my heart I can’t walk like I used to, or I have to walk slowly.
Sense of isolation		0201	III	Before COVID-19, even if I was already feeling unwell, I would go out, I would go to places, on Sundays I would go to the movies. Now with Covid, since we spent more than a year without being able to go out, I feel depressed.
		306	III	I miss having the independence to be able to meet and catch up with my friends. Now I ride an electric scooter, but I can’t spend much time away from home because I can’t get up from the scooter.
		303	III	I have always been very social. Now I don’t see my friends because I can’t keep up when they meet up for a walk.
**Domain 2. The new role of informal caregiving**				
		203	II	He no longer dares to go out alone, he always has to go with me. I have also learned how to give him the rescue medication and I am in charge of keeping track of the visits and phone calls with the hospital. [Caregiver]
		206	II	I have a notebook in which every morning I write down her weight, meals, and medications. I always carry it with me during medical visits. [Caregiver]
		301	III	My sisters and I have had to organize ourselves, and I have had to bring him/her food home, making sure s/he eats and that s/he eats well. Lately s/he doesn’t have an appetite, so I can tell how s/he is doing from the lunch boxes she leaves. [Caregiver]
		306	III	It is difficult for me to have to depend on my wife. I have always been very independent and now I depend on my wife for everything, like washing or dressing. [Patient about her caregiver]
		401	IV	She has already been admitted several times and I have learned to know when to call an ambulance. The last time I called because I noticed that my mother was moving as if she was in slow motion. I asked her to touch her nose with her hand, but she was asleep with her hand up, she didn’t have the strength to open her eyes. My mother never complains or says anything when they call her … So since I have telecare [remote care device], as soon as I see something strange, the first thing I do is press the button. When they arrived, she had arrhythmia and was taken to the hospital. At any rate, I am not a nurse and I am afraid that at some point something could go wrong. [Caregiver]
		401	IV	I also have a house and a family, but I also have my mother. That’s why I had to appoint a caregiver who would go every day, otherwise I would have been overworked from Monday to Friday. [Caregiver]
**Domain 3. The value of multidisciplinary care**				
The new relationship with primary care		101	I	Partly due to the pandemic, when I phone my primary care physician, they rarely ever pick up. My heart problem was detected by the anesthesiologist who was getting me ready for a cataract operation. He saw something strange with the coagulation and referred me to the cardiologist at the hospital. If it were up to outpatient care, I’d still be waiting.
		204	II	With many of these health professionals, we have no communication. That’s why I have to say no to almost everything. The two of us are here to take care of each other, I take care of him and he takes care of me because we are both sick. [Caregiver]
		201	II	I overwhelm my primary care physician, the poor thing. I start by telling her one thing and then another, and another, and another. I say that I overwhelm her because there are so many things. When it’s not for low blood sugar, then it’s for an embolism, a heart problem, a kidney problem or high blood pressure. I say that it is a lot for them to take on in the outpatient facility.
		201	II	Neither my doctor nor the outpatient nurse know how to monitor my blood sugar. They tell me that I better monitor it myself because I know myself better than they do. Before, they gave me more guidance. They told me “have a little less, have a little more”. Since being admitted, not anymore.
		301	III	When one has a disease, they usually seek out the cause in order to treat it. With my mother, the specialists do look into it more, but the primary care ones don’t look into it. They haven’t seen what causes everything that happens to my mother. [Caregiver]
		303	III	The relationship with primary care has been really bad. When I call, I get music and then they hang up on me. I don’t feel like I can count on them if I get worse.
		401	IV	Her family doctor isn’t really paying attention and doesn’t know about it. The ones closest to her are the paramedics and nurses who come to the house, who are more vigilant. But when I talk to her doctor and ask her about something that has happened, she never knows what’s going on. I get the impression that they don’t communicate with each other. [Caregiver]
Provision of clinical support from HCPs		101	I	During the first visit [in the cardiology unit] they explained to us that the fatigue could be due to several causes. So the first thing was to look at her medication and adjust it. The internal medicine specialist taught her how to take the thyroid medication that we knew she was taking incorrectly. The cardiologist did the ultrasound right then and there and explained to us about the deficiency, and the nurse told her how to weigh herself and measure her blood pressure. They told us that from then on they were going to take her directly from the hospital for a follow-up. This gave me a lot of peace of mind. [Caregiver]
		204	II	Now he is much more monitored than before because this team of cardiologists has taken her on and they have fine-tuned her treatment. We spent an hour with these doctors who visited with her, and I liked it and thought they treated her well. You don’t have to go from one specialist to another. They see everything that is heart-related. [Caregiver]
		202	II	Both the cardiologist and the nurse talk to me a lot about what is going on with me and make recommendations about medication and diet.
		202	II	They have treated me wonderfully, they even called me from the hospital to see how I am doing.
		304	III	My doctor has a very serious personality, but she is wonderful, a very good person. It is very difficult for a transplant recipient to spend several years like this. I don’t see her as my doctor, I see her as a family member.
		302	III	The primary care physician is very nice, but since they took me to cardiology, I haven’t seen him and I haven’t continued with him. Well, they are all nice, very attentive. The nurses too. I have no complaints.
		302	III	I trust the doctor a lot, I ask him to please treat me as if I were his mother.
Provision of nursing care by a registered nurse		201	II	This week, the hospital nurses have already called several times. I feel that they care about me, they usually call every week to see how I am doing.
		203	II	The nurse always calls us. We have even done video calls from our house in the countryside, since I took him there in spring, and well, he had wonderful views sitting there at the door of his farmhouse, doing a video call with the nurse. I told him, you won’t have complaints about the care. [Caregiver]

**Table 4:** Patient-reported outcome measures by NYHA classification.

	NYHA I–II (*n* = 11)	NYHA III–IV (*n* = 9)	All NYHA (*n* = 20)
**EQ-5D-5L**			
**EQ-ED-5L, patients reporting no problems, *n* (%)**			
Mobility	4 (40%)	0	4 (21%)
Self-care	6 (60%)	4 (44.4%)	10 (53%)
Usual activities	2 (20%)	1 (11.1%)	3 (16%)
Pain/discomfort	6 (60%)	0	6 (32%)
Anxiety/depression	5 (50%)	4 (44.4%)	9 (47%)
**EQ-ED-5L, patients reporting slight or moderate problems, *n* (%)**			
Mobility	5 (50%)	6 (66.6%)	11 (58%)
Self-care	3 (30%)	3 (33.3%)	6 (32%)
Usual activities	7 (70%)	4 (44.4%)	11 (58%)
Pain/discomfort	2 (20%)	7 (77.8%)	9 (47%)
Anxiety/depression	5 (50%)	3 (33.3%)	8 (42%)
**EQ-ED-5L, patients reporting severe or extreme problems, *n* (%)**			
Mobility	1 (10%)	3 (33.3%)	4 (21%)
Self-care	1 (10%)	2 (22.2%)	3 (16%)
Usual activities	1 (10%)	4 (44.4%)	5 (26%)
Pain/discomfort	2 (20%)	2 (22.2%)	4 (21%)
Anxiety/depression	0	2 (22.2%)	2 (11%)
**EQ-ED-5L global, mean (SD)**			
Index value	0.68 (0.26)	0.34 (0.46)	0.52 (0.40)
Quality of life visual analogue scale	74 (13.56)	51.6 (23.4)	63.42 (23.05)
**KCCQ, mean (SD)**			
Physical limitation	62.42 (33.04)	31.25 (29.28)	47.65(33.04)
Symptom stability	67.50 (23.72)	52.78 (26.35)	60.53 (25.43)
Symptom frequency	73.96 (16.56)	41.44 (28.29)	58.55 (27.77)
Symptom burden	71.67 (16.66)	47.22 (23.94)	60.09 (23.83)
Self-efficacy	82.50 (24.44)	86.11 (14.58)	84.21 (19.91)
Quality of life	59.17 (24.36)	28.70 (29.50)	44.74 (30.46)
Social limitation	68.75(24.79)	24.77 (34.68)	47, 92 (36.75)
**KCCQ, global scores, mean (SD)**			
Overall summary	65.79 (21.59)	32.26 (26.27)	59.91 (28.90)
Clinical summary	67.61 (22.13)	37.79 (23.40)	53.49 (26.88)
Total symptom	72.81 (16.31)	44.33 (25.11)	59.32 (25.03)
**PGIS, patient’s response, *n* (%)**			
No symptoms, very slightly, slightly	7 (70%)	5 (55.5%)	12 (63%)
Moderate, intense, extreme	3 (30%)	4 (44.4%)	7 (37%)
**IEXPAC mean (SD)**			
Caregiver *	5.38 (2.65)	5.79 (2.55) *	5.55 (2.54)
Conditional questions	6.71 (2.25)	6.25 (3.75)	6.46 (2.99)
Patients	6.75 (1.08)	6.46 (1.80)	6.61 (1.43)
Conditional questions	7.97 (2.36)	5.68 (2.36)	6.60 (2.52)

* There are 2 patients that do not have caregiver and therefore not included in analysis (NYHA II: 201) & (NYHA IV: 208).

### 3.4. Complementarity and Discrepancies Between Qualitative and Quantitative Findings

Complementarity examples:

The difference in the experience of patients with different NYHA classes was captured in both the interviews and the descriptive analysis of PROMs’ answers (Table 4). Patients with NYHA class I/II tended to report slight/moderate problems with mobility and usual activities in the EQ-ED-5L questionnaire, while patients with higher NYHA classes tended to report moderate/severe problems related to pain or discomfort. In the KCCQ, patients with lower NYHAs reported better scores regarding most areas than patients with greater severity, especially physical limitations, symptoms frequency, self-efficacy, QoL, and social limitation. This coincided with the data gathered through the interviews.

Furthermore, the severe decline in functional ability and the need for assistance were well-documented in the qualitative data. However, while the quantitative data showed moderate scores in mobility and QoL, the emotional impact described (e.g., loss of independence and increased need for help) suggests a deeper level of distress that may not be fully conveyed by the numerical scores alone. For instance, patient 401 (NYHA IV) said, “It has gotten worse and worse, now my daughters have sent me a lady to come and help me at home. For the past year I can no longer do things alone, neither get dressed nor clean myself.” Moreover, patient 305 (NYHA III) said, “For me, quality of life is being independent. I suffer because now I am not able to go to the supermarket, make my bed, prepare meals or clean the house.” Thus, the complementary nature of qualitative data contributed to a more comprehensive understanding of overall QoL.

Discrepancy examples:

Self-efficacy: KCCQ Self-efficacy scores were high for both NYHA I-II (82.50) and NYHA III-IV (86.11) patients; however, many patients expressed feeling unprepared or unable to manage their condition independently, particularly those in higher NYHA classes. Patient 305 (NYHA III) said “For me, quality of life is being independent. I suffer because now I am not able to go to the supermarket, make my bed, prepare meals or clean the house.”

Self-care abilities: In total, 44.4% of NYHA III-IV patients reported no problems with self-care on EQ-5D-5L; however, they expressed significant difficulties with self-care during the interviews. For instance, patient 401 (NYHA IV) stated, “For the past year I can no longer do things alone, neither get dressed nor clean myself.”

Anxiety and depression: In total, 44.4% of NYHA III-IV patients reported no problems with anxiety/depression on EQ-5D-5L; however, multiple patients expressed feelings of low motivation, anxiety, and concerns about physical impairment and social isolation. For instance, patient 303 (NYHA III) mentioned, “There are days that I feel very low energy, without motivation. It’s not that I’m depressed, but just mad at myself because I’m not feeling well.”

Symptom stability: Furthermore, this patient’s expression of fluctuating energy levels and frustration reflects an emotional and psychological burden that may not align directly with the KCCQ scores, which provide an average measure of symptom burden but may miss day-to-day variations and emotional impacts. KCCQ symptom stability scores were relatively high for both NYHA I-II (67.50) and NYHA III-IV (52.78) patients; however, patients across all NYHA classes expressed concerns about unpredictable symptom fluctuations. For example, patient 303 (NYHA III) said, “Today for example I feel fine, tomorrow I don’t know.”

Pain and discomfort: Only 22% of NYHA III-IV patients reported severe/extreme problems with pain/discomfort on the EQ-5D-5L; however, interviews revealed patients’ significant concerns about physical decline and discomfort in their daily lives.

Social limitations: The KCCQ social limitation scores for NYHA I/II patients indicated fair to good/excellent health status; however, interviews revealed a sense of isolation and loss of agency (e.g., inability to travel), even among patients with lower NYHA classes.

PROMs were a valuable tool for gaining an overview of the perspectives of patients on their health status and disease expectations in the previous weeks or the same day of questionnaire compilation. They were also used as conversation starters in the qualitative interviews, helping to introduce sensitive topics. Nevertheless, the inconsistencies between PROMs results and qualitative interviews’ data suggest that some complex emotional, social, and physical states are not always adequately captured by quantitative tools. For example, questions that refer to specific timeframes or include non-specific examples in questionnaires may result in incomplete responses. This underscores the importance of combining quantitative and qualitative methods to capture the contextual nuances of patients’ experiences.

## 4. Discussion

This study examined 19 patients’ and 17 caregivers’ experiences with HFpEF to gain insights into the impact of the disease on QoL, relationships and interactions with HCPs and caregivers, and cognitive, emotional, and functional needs. Three main themes were identified as factors for improving patients’ QoL and care: the impact of HFpEF on QoL, new informal caregiving roles, and the increasing value of multidisciplinary care. Moreover, it was illustrated that the generic QoL-related questionnaires did not fully capture HF patients’ disease burden and that discrepancies existed between patients’ and caregivers’ perceptions of the impact of the disease on QoL.

According to HFpEF patients’ reported life experiences, a linear decline in QoL was observed as severity based on NYHA class increased. Patients went from a sense of normalcy to a sense of less control over their situation. These findings are sensible considering HF is chronic and worsens over time. This evolution was also reflected in the scores from the disease-specific and generic PROM questionnaires. A higher percentage of NYHA class III/IV patients reported severe/extreme difficulties with mobility, self-care, usual activities, pain/discomfort, and anxiety/depression compared to NYHA lower classes. Similarly, KCCQ scores in all of the descriptive dimensions were better in NYHA I/II patients compared with those in NYHA class III/IV, congruent with the previous literature.

The deterioration of HFpEF patients’ QoL, especially as the disease worsens, leads to an increase in attention and support from caregivers, who feel HF patients are no longer prepared to face the illness alone. Informal caregivers are usually family members and coordinate care with other family members, attend medical appointments, and provide daily assistance, many of whom have comorbidities [47,48]. This puts stress on caregivers, who combine care with their own responsibilities while also providing patients emotional support [47,48,49,50,51,52,53]. Caregivers’ experiences were more critical than patients’ and highlighted a greater degree of deficiencies in medical care processes and associated caring costs. This trend was reflected in interviews and caregivers’ low scores in the IEXPAC questionnaire, which highlights the need for caregivers to have more knowledge about HFpEF and feel more supported by HCPs so that their needs are addressed and they can best support HFpEF patients.

The third theme revealed the increasing value of multidisciplinary care. HF patients who typically have strong relationships with PC physicians experienced a sense of abandonment due to the pandemic. Nevertheless, HFpEF patients increasingly relied on specialists because of expert attention. This led advanced practice nurses to take greater responsibility in their care process. To the best of our knowledge, there is no other research that has identified this trend in the Spanish context or in the post-pandemic landscape.

Education from medical specialists and advanced practice nurses also allowed patients to be more aware of the disease and its limitations, consistent with previous studies demonstrating how specialized interventions improved disease management and experience [54,55,56]. These findings emphasize the importance of patients’ feelings of abandonment by PC physicians, which are particularly worrying because of potential consequences for patients’ health and impacts on patient services/costs. It has been reported that well-integrated multidisciplinary care with strong collaboration between hospitals and PC significantly decreases readmissions for HF and mortality [57]. However, despite awareness of the impact of such programs in the broader HF space, to the best of our knowledge, there is limited research on multidisciplinary approaches specific to HFpEF management [58,59]. This article contributes to the small body of research that calls for such programs. Therefore, in the post-pandemic landscape, transition to more integrated management (e.g., specialized/PC) is needed to reduce HF burden.

Most clinical trials and observational studies have mainly focused on HFrEF, resulting in a lack of awareness of HFpEF among clinicians and society [60,61]. As occurs with HFrEF patients, HFpEF patients experience a significant deterioration in their QoL as the disease worsens, but the low visibility of these patients adds more burden to their already impaired QoL [7]. The results of this study have shown that the disease experience of patients with HFpEF and their caregivers is similar to that of patients with HFrEF [5]. This is relevant given that they focus on considerably different epidemiological populations. This study therefore addressed an important gap in the evidence by providing valuable, complementary information regarding patients’ QoL experience regarding HFpEF. Understanding these attitudes has strong implications for clinical care in multidisciplinary settings, favoring the addition of advanced practice nurses, better access of HFpEF patients to PC, and, therefore, a continued care process across HCPs.

Finally, some discrepancies were found between the qualitative and the descriptive quantitative data regarding self-efficacy, self-care abilities, anxiety and depression, symptom stability, pain and discomfort, and social limitations. A few studies have compared data reported via PROMs and interviews in HF [5,62,63]. For instance, Davis et al. found that the KCCQ could not adequately capture how HF affected key health components, such as social relationships and mental health, among racially diverse, low-income patients [62]. Furthermore, Gwaltney et al. found that pain was not adequately captured in PROM instruments assessing HF [63], while Rubio et al. highlighted discrepancies in the patient’s self-caring strategies, perception of autonomy, level of empowerment, and perception of past and present experiences with the disease [5].

It is possible that the semi-structured interview allowed for a deeper explanation of patients’ experiences with questions that captured all factors that impact daily life with HFpEF, such as understanding emotional conditions and other important sentiments related to perception of past encounters, autonomy, empowerment, and self-care strategies. Therefore, the current study raises questions regarding how to appropriately assess HFpEF patients’ QoL. This is especially true for this population, which shows that results of previous studies in HF patients should not necessarily be extrapolated to these patients as they have not been evaluated for patients with preserved cardiac function. Future research should focus on the subjectivity that comes with measuring QoL in patients with HFpEF and the identification of effective and coordinated medical strategies to improve the QoL burden in the HFpEF population.

### Strengths and Limitations

This study has some limitations. First, the sample size, although robust for qualitative studies to capture diverse experiences across disease stages, may not represent the full heterogeneity of HFpEF patients and caregivers. Second, questions addressing earlier stages of the patients’ journey may have led to recall bias. Third, the sample was not stratified according to frequent comorbidities, which may have brought insightful perspective on potential experiences between different types of HFpEF patients. However, the ethnographic approach of this study has provided a rich understanding of the experiences of patients with HFpEF and their caregivers, filling a gap in the literature on these type of patients in Spain and contributing to the limited qualitative research on HFpEF globally. Future directions include larger-scale studies and longitudinal research to track changes over time, building upon day-to-day variations captured in this study.

## 5. Conclusions

The study identified three main themes and sub-themes: (1) the impact of HFpEF on QoL, characterized by a sense of isolation, autonomy loss, and fear of decline/progression; (2) new roles of informal caregiving, including gender differences and spouses and children; and (3) the increasing value of multidisciplinary care, characterized by new relationships with PC physicians, the provision of specialized support from HCPs, and the provision of nursing care by registered nurses. Qualitative data were supported by a general trend of worsening QoL on quantitative measures as HF progressed, despite quantitative measures not fully capturing the burden. Qualitative data further captured discrepancies between patients’ and caregivers’ QoL perceptions. These additional insights could serve to better tailor QoL questionnaires. More generally, questionnaires’ results may become more accurate if specifically designed for HFpEF patients or if they encompass broader timeframes beyond the six months previous to the interview, as in the case of IEXPAC. Furthermore, future mixed-methods studies could further contribute to the analysis of the broader impact of the disease. The main goal of this article was to identify unmet needs of patients and caregivers and to provide specific insights that could inform the customization of care.

## Figures and Tables

**Table 1:** Demographic and clinical characteristics (*n* = 19).

Participants (*n* = 19)	*N*	%
Age, years, mean (SD)	80 (7)	
Range (minimum–maximum)	67–92	
Gender, male, *n* (%)	8	42
Time since diagnosis, years, mean (SD)	3.3 (5.7)	
Recent diagnosis (less than 2 months), *n* (%)	2	10
NYHA classification, *n* (%)		
I	4	21
II	6	32
III	7	37
IV	2	10
LVEF, mean (SD)	64 (7.3)	
Range (minimum–maximum)	50–78	

**Table 2:** Social characteristics of patients (*n* = 19).

Verbatim Code	NYHA	Age	Gender	Educational Level	Marital Status	Main Caregiver	Number of Cohabitants *
101	I	80	Female	Elementary	Widow	Daughter	0
103	I	76	Male	Elementary	Married	Wife	1
104	I	80	Male	University	Married	Wife	2
105	I	71	Female	Elementary	Married	Husband	2
201	II	67	Female	Elementary	Married	Husband	1
202	II	86	Male	Elementary	Married	Wife	1
203	II	86	Male	Elementary	Married	Wife	2
204	II	77	Male	Elementary	Married	Wife	1
205	II	67	Male	Elementary	Married	Wife	1
206	II	92	Female	Elementary	Widow	Daughter	1
301	III	88	Male	Elementary	Widow	Daughter	0
302	III	82	Female	Elementary	Widow	Son	1
303	III	82	Female	Elementary	Single	Niece	0
304	III	72	Female	Elementary	Single	Brother	0
305	III	85	Female	Elementary	Widow	Daughter	0
306	III	77	Male	Elementary	Married	Wife	1
307	III	87	Female	Elementary	Widow	Son	1
401	IV	83	Female	Elementary	Widow	Daughter	1
402	IV	85	Female	University	Widow	Son	0

(*) Total number of cohabitants excluding the participant.

## Data Availability

The data that have been used are confidential, as established by the Ethics Committee and the informed consent form signed by participants.
